# Modafinil as a Catecholaminergic Agent: Empirical Evidence and Unanswered Questions

**DOI:** 10.3389/fneur.2013.00139

**Published:** 2013-10-07

**Authors:** Jonathan Wisor

**Affiliations:** ^1^Department of Integrative Physiology and Neuroscience, Sleep and Performance Research Center, Washington State University, Spokane, WA, USA

**Keywords:** modafinil, amphetamines, cocaine, dopamine transporter, monoamines, sleep homeostasis, addiction, wake-promoting therapeutics

## Abstract

Modafinil, in its two clinical formulations (Provigil^®^ and Nuvigil^®^), is a widely prescribed wake-promoting therapeutic agent. It binds competitively to the cell-membrane dopamine (DA) transporter and is dependent on catecholaminergic (dopaminergic and adrenergic) signaling for its wake-promoting effects. The clinical spectrum of effects for modafinil is distinct from the effects seen with other catecholaminergic agents. Relative to other commonly used agents that act through catecholaminergic mechanisms, modafinil has a relatively low abuse potential, produces wakefulness with an attenuated compensatory sleep recovery thereafter, and does not ameliorate cataplexy in narcolepsy. These clinically relevant phenomenological differences between modafinil and agents such as amphetamines and cocaine do not eliminate catecholaminergic effects as a possible mediator of its wake-promoting action; they merely reflect its unique pharmacological profile. Modafinil is an exceptionally weak, but apparently very selective, DA transporter inhibitor. The pharmacodynamic response to modafinil, as measured by DA levels in brain microdialyzate, is protracted relative to other agents that act via catecholaminergic mechanisms. The conformational constraints on the interaction of modafinil with the DA transporter – and probably, as a consequence, its effects on trace amine receptor signaling in the catecholaminergic cell – are unique among catecholaminergic agents. These unique pharmacological properties of modafinil should be considered both in seeking to thoroughly understand its putatively elusive mechanism of action and in the design of novel therapeutic agents.

## Introduction

Modafinil was originally introduced in the clinical literature as a wake-promoting agent in 1988 (1). Modafinil was first approved by the US Food and Drug Administration (FDA) in 1998 and marketed as the racemic mixture of R- and S-enantiomers (2) and later as a formulation containing only the R-enantiomer, which is pharmacokinetically distinct from the S-enantiomer in humans (3) as described later (4). It has been viewed throughout its history (5–7) as a “novel” wake-promoting therapeutic, and apparently is still viewed in the same manner to this day (8). The fact that this mainstream therapeutic agent is still thought of as novel presumably stems from some unique pharmacological and clinically relevant properties of modafinil relative to other wake-promoting agents. The purpose of this review is to summarize the known pharmacological properties of modafinil and to explain the unique clinical responses to modafinil in the context of these properties. In so doing, this review will expose some unanswered questions regarding the mechanism of action of modafinil and will offer insights relevant to the discovery and preclinical development of other novel (in a stricter sense) wake-promoting agents.

## Modafinil Binds to the Dopamine Transporter and is Dependent on Catecholaminergic Signaling for Its Wake-Promoting Effects

Like many compounds, modafinil was found to be clinically useful long before its pharmacological target was known. Still, as with any new wake-promoting agent, a number of potential targets came to mind in the search for its mechanism of action. Among the potential targets for modafinil were the cell-membrane monoamine transporters. These monoamine-selective transporters serve, in a non-selective fashion, to clear the monoamines dopamine (DA), noradrenaline (NE), and serotonin (5-HT) from the extracellular space surrounding the neurons that release them. The transporters are named for the neurochemical identity of the cells that express them at the highest levels – the dopamine transporter (DAT), the noradrenaline transporter (NET), and the serotonin transporter (SERT). Despite this nomenclature, they are not truly selective for their namesake neuromodulators. For instance, the Michaelis constant (*K*_m_) for DA uptake by the NET is lower than the *K*_m_ for NE uptake by the NET [at least in genetically engineered cells expressing NET under an exogenous construct (9)], indicating a higher affinity for DA. This fact, and the promiscuity among catecholaminergic (dopaminergic and adrenergic) receptors in responding to both DA and NE, may lead to some confusion with regard to modafinil’s mechanism of action (see below).

At the time when modafinil’s wake-promoting effect was discovered, various agents that bind to and inhibit the activity of monoamine transporters, such as cocaine and amphetamines, were known to also promote wakefulness. Thus, it was reasonable to hypothesize that modafinil might act through monoamine transporter inhibition to produce wakefulness. The first indication that modafinil binds competitively to a monoamine transporter, specifically DAT, came in 1994 (10). In that study, modafinil competitively displaced the binding of radiolabeled (−)-2-β-Carbomethoxy-3-β-(4-fluorophenyl)tropane (abbreviated β-CFT, also known as WIN 35,428), a known DAT ligand, in extracts of a tissue enriched for DAT, the striatum, with a concentration that inhibits 50% (IC_50_) value of 3.19 μM. The ability of modafinil to displace SERT and NET ligands was investigated in the same study and no effect was detected.

Competitive binding of modafinil to the DAT has been replicated in human embryonic kidney HEK293 cells transfected with DAT-encoding genetic constructs. In these studies, racemic modafinil displaced WIN 35,428 with inhibition constant [*K*_i_] values of 2.1 μM (11) and in a separate study 2.3 μM (12). The latter study additionally reported enantiomer-specific *K*_i_ values, which were threefold higher for S-modafinil (2.5 μM) than R-modafinil (0.78 μM). These data are summarized in Table 1.

**Table 1:** **Affinity of modafinil compared to other dopamine reuptake inhibitors**.

Agent/drug	DAT-binding affinity: competitive displacement of ^3^H-WIN 35,428 (*K*_i_)					DAT functional assays: inhibition of ^3^H-DA reuptake (IC_50_)		
	(10)^a^	(13)^b^	(11)^c^	(14)^d^^,^^f^	(12)^e^	(12)^e^	(14)^d^	(15)^c^
Modafinil (nM)	1930	3800	2143	4800	2300	13,000	4043	6390
Cocaine (nM)	46.2	–	163.6	187	450	230	487	–
Methylphenidate (nM)	–	–	21.2	–	–	–	–	25.4
Bupropion (nM)	383	310	319.5	–	–	–	–	1088
Nomifensine (nM)	36.9	44	–	–	–	–	–	–
β-CFT (nM)	–	–	15.4	–	–	–	–	–
GBR 12909 (nM)	–	–	53.2	12	–	–	4.3	–
Benztropine (nM)	–	–	75.3	–	–	–	–	213

β-CFT, (−)-2-β-Carbomethoxy-3-β-(4-fluorophenyl)tropane (also known as WIN 35,428); DAT, dopamine transporter; IC_50_, concentration that inhibits 50%; *K*_i_, inhibition constant.

^a^Guinea pig striatal membranes;

^b^Canine DAT;

^c^HEK293 cells transfected with human DAT;

^d^Rat brain synaptosomes;

^e^COS7 cells transfected with human DAT;

^f^Competitive displacement of ^125^I-RTI-55.

Other studies showed that high concentrations of modafinil (relative to known DAT inhibitors) block DA uptake by cell lines stably transfected with the DAT. IC_50_ values for modafinil in these *in vitro* assays range from 4.0 to 13 μM (12, 14, 15). IC_50_ values for DA uptake inhibition are enantiomer-specific and twofold higher for S-modafinil (8.7 μM) relative to R-modafinil [4.0 μM; (12)]. Additionally, it has been demonstrated in positron emission tomography (PET) studies that modafinil causes the displacement of the DA-receptor ligand raclopride and the DAT ligand cocaine in the human brain (16). Similarly, modafinil displaces WIN 35,428 in the non-human primate brain (15). Displacement of a DA-receptor PET ligand is not necessarily evidence of direct binding of modafinil to the receptor. The displacement of a DA-receptor ligand by modafinil is likely to be a consequence of elevated extracellular DA concentrations, a known consequence of modafinil administration (12, 14, 17), rather than binding of modafinil to the DA receptor.

There is some evidence that modafinil binds to the NET in addition to the DAT. In PET studies, modafinil displaced the binding of radiolabeled NET ligand [^11^C]MeNER in the monkey thalamus (15). In cultured HEK293 cells transfected with human NET, modafinil inhibited NE uptake with an IC_50_ value of 35.6 μM (15). However, in other studies where NET binding or effects on NET activity were assessed *in vitro* (10, 12–14, 18), modafinil was found to be devoid of interactions with NET. Furthermore, the absence of therapeutic efficacy for modafinil in treating cataplexy in narcoleptic humans (19) or animal models (20, 21) makes it unlikely that modafinil is a functional NET inhibitor *in vivo*. NET inhibitors are very effective as anti-cataplectic agents (22), whereas DAT inhibitors are not (13).

If modafinil is a DAT inhibitor, and the blockade of DATs by modafinil is central to its wake-promoting effects, several predictions can be made and tested experimentally. First, one would expect that genetic ablation of the DAT would nullify the wake-promoting effect of modafinil if indeed this is the site of action. In fact, the wake-promoting effect of modafinil is abolished in mice genetically deficient for DAT (17). Second, one would expect modafinil administration to elevate extracellular DA concentrations *in vivo*, and it does. Modafinil administration increased extracellular DA concentrations in the caudate nuclei of narcoleptic dogs by twofold relative to baseline (17) and in the nucleus accumbens of mice (12) and rats (14, 23) by approximately two to threefold relative to baseline. Despite enantiomeric differences in DAT ligand displacement and DA uptake inhibition mentioned previously, R-modafinil and S-modafinil were equally effective in elevating microdialyzate DA concentration in the mouse nucleus accumbens at systemic doses of 30–300 mg/kg (12).

If elevation of dopaminergic tone underlies the wake-promoting effect of modafinil, one would expect therapeutic responses to modafinil to be dependent on the activation of DA receptors. Either a D1 or D2 DA antagonist is sufficient to block the wake-promoting effects of low wake-promoting doses (≤45 mg/kg) of modafinil in wild-type mice. In D2-deficient mice, the arousal response to high-dose (90–180 mg/kg) modafinil is attenuated relative to the wild-type response in the absence of pharmacological receptor blockade and is abolished by D1-antagonist application (24). Are these DA receptor-dependent effects of modafinil necessarily secondary to DAT blockade, or could they be indicative of agonist activity at D1 or D2 receptors? A single publication reported, in native rat striatal homogenates, that R-modafinil, but not S-modafinil, displaces the D2 receptor ligand domperidone with nanomolar potency (25). This result contrasts the work of others showing no displacement of D2 ligands by racemic modafinil at concentrations less than 10 mM [sulpiride in Ref. (10); *N*-methylspiperone in Ref. (14)]. Further work, including measuring the effects of R-modafinil and S-modafinil separately in D2-deficient mice, may help to clarify whether binding to the D2 receptor contributes to the wake-promoting effects of the R-enantiomer, specifically.

Collectively, these data make a compelling case for the concept that the wake-promoting effects of modafinil are mediated by its interaction with the DAT and elevation of dopaminergic tone.

## Putative Non-Dopaminergic Effects of Modafinil *In vivo* may be Secondary to DAT Binding

Neural signaling systems other than DA and its receptors have been implicated in the brain’s response to modafinil, but the other responses could be triggered secondary to elevated concentrations of brain DA. For instance, the alpha-1 adrenergic antagonist prazosin prevented modafinil-induced, behaviorally defined nocturnal awakenings in monkeys (26) and electroencephalogram (EEG)-defined wakefulness in cats (27). Two DA-dependent mechanisms might explain this linkage of modafinil’s wake-promoting effect to adrenergic receptors. As a ligand for alpha-1 adrenergic receptors, DA is very nearly equipotent with NE (28). So the elevation of extracellular DA concentrations by modafinil should be expected to directly activate adrenergic receptors wherever they lie in close proximity to DAT-bearing dopaminergic terminals in the brain.

Additionally, modafinil elevates NE concentrations in both the prefrontal cortex and the hypothalamus (29). This response can be explained by a D1 receptor-mediated effect, as DA infusion into the prefrontal cortex elevates extracellular NE concentrations in a D1 receptor-dependent manner (30). Whether the adrenergic component of the response to modafinil is a direct effect of DA binding to adrenergic receptors or secondary to D1 receptor-induced elevation of NE, the role for alpha-1 adrenergic receptors does not violate the conceptual framework of modafinil as a DAT blocker.

Similar logic applies to other neurotransmitter responses to modafinil. Modafinil precipitates a decrease in concentrations of gamma-aminobutyric acid (GABA) in microdialyzates from various brain areas (31–33). This effect, at least in the cerebral cortex, is dependent on catecholaminergic signaling, as it is attenuated by the catecholaminergic toxin 6-hydroxy-DA (32). Furthermore, D1 agonists precipitate a reduction in GABA concentrations in cortical microdialyzates (34).

A similar line of reasoning applies to glutamate. Modafinil promotes an increase in extracellular glutamate concentrations in the striatum (31) and the hippocampus (35). The DA agonist apomorphine promotes an increase in extracellular glutamate concentrations in the striatum (36, 37), although the interactions of DA in this region are admittedly complex and not entirely consistent across experiments (38). DA itself promotes an increase in glutamate release in the hippocampus (39). The increase in glutamate release in these areas after modafinil administration may, thus, be secondary to elevated extracellular DA. This logic could be applied to the other transmitter systems known to be affected by modafinil (40).

So yes, modafinil has effects on adrenergic, GABAergic, and glutamatergic transmission, but all of these effects can be explained by its known pharmacology as a DAT blocker. Still, though it is one thing to argue that these responses are secondary to elevated dopaminergic tone, it is another to ascertain that they are. To do so, one would have to show that each of these hypothesized secondary responses is abolished in DAT-deficient animals and in wild-type animals treated with a panel of DA-receptor antagonists. Given the preponderance of evidence for a dopaminergic mechanism, these experiments should be a top priority for anyone seeking to document any putative non-dopaminergic mechanism of action.

## Why Do Modafinil and Other Commonly Used (and Abused) DAT Inhibitors have Distinct Effects on Clinically Relevant Measures?

The effects of modafinil on sleep and sleep disorders are distinct from those of methamphetamines. Sleep loss induced by sleep deprivation is followed by a change in EEG parameters, including increased time spent asleep, increased duration of individual sleep episodes, elevated slow-wave activity in the EEG, and decreased numbers of awakenings. This constellation of changes, sometimes referred to as hypersomnolence or sleep rebound, has been observed in experimental rodents (41) and humans (42, 43) alike.

The occurrence of hypersomnolence after sleep loss is taken as evidence that sleep is a homeostatic process, and that homeostatic sleep need builds as a function of time spent awake (44). In this context, the effects of modafinil on sleep homeostasis differed from those of methamphetamine when the two were compared. Whereas hypersomnolence occurred after methamphetamine-induced wakefulness in rats, it was not detected after an equivalent wake-promoting dose of modafinil (6, 45). One interpretation for this difference is that the two compounds have distinct effects on the biological substrates for homeostatic sleep need. Specifically, modafinil might decelerate, or methamphetamine might accelerate, the rate at which sleep need accumulates during wakefulness. Regarding the first of these two possibilities, a head-to-head comparison of the severity of hypersomnolence subsequent to modafinil-induced wakefulness and sleep deprivation-induced wakefulness in mice found no difference between conditions (46). Similarly, in human subjects, administration of modafinil during enforced wakefulness did not, relative to placebo, attenuate the increase in slow-wave activity that occurred in subsequent sleep (47, 48). Therefore, modafinil does not decelerate the rate at which sleep need accumulates during wakefulness.

It is possible that methamphetamine accelerates the accumulation of sleep need; this effect may be due to its activity as a disruptor of NET and SERT. One might hypothesize that the direct action of methamphetamine on serotonergic and noradrenergic terminals (49) contributes to methamphetamine-induced hypersomnolence. While the activity of 5-HT-producing cells of the raphe- and NE-producing cells of the locus ceruleus is greatest in wakefulness and strongly reduced during sleep, both NE (50) and 5-HT (51–53) promote homeostatic sleep drive. The direct perturbation of noradrenergic and serotonergic terminals by amphetamines may contribute to the hypersomnolence that they cause. Assessment of the effects of amphetamines on sleep in NET- and SERT-null mutants might address this possibility. Attenuation of amphetamine-induced hypersomnolence by knockout of either NET or SERT would confirm that they contribute to amphetamine-induced hypersomnolence.

Measures of gross locomotor behavior have long been applied to measure the psychostimulant effects of cocaine, amphetamines, and other DAT-binding agents. Locomotor effects of modafinil have been compared and contrasted to those of cocaine and amphetamines and the data are not consistent across studies. Acute administration of modafinil increases locomotor activity in rodents (23, 54, 55), much like cocaine and amphetamines. However, electroencephalographic studies in rats demonstrated that the intensity of locomotor activity (amount of locomotor activity per hour of wakefulness) in a home cage environment was not increased by modafinil relative to vehicle controls, in contrast to d-methamphetamine which elevated the amount of locomotor activity per hour of wakefulness (6). Similarly, video monitoring of Syrian hamsters revealed that modafinil increased time spent in quiet wakefulness (“arousal without ambulation, head up, eyes open”), but not wake with ambulation, relative to a vehicle injection (56). Cocaine and methamphetamine effects on behavior were not measured in the latter study. Hence, the results cannot be taken as evidence for a unique effect of modafinil; there may be a species-specific response to DAT inhibitors, for instance. One study reported a “non-amphetaminic mechanism” for modafinil based on the absence of stereotyped climbing (“repetitive locomotor activity”) in modafinil-treated rodents (57). However, at least two other studies did report stereotypy after modafinil administration [“repetitive movements” in Ref. (14) and “repetitive oral movements, such as gnawing, biting, and sniffing” in Ref. (58)]. Inconsistency across studies aside, none of these behavioral studies demonstrated a pharmacological mechanism of action for modafinil.

Both amphetamines and cocaine produce locomotor sensitization, in which the amount of induced locomotor activity increases with repeated daily administration over time. Rats exhibit locomotor sensitization to repeated daily modafinil administration at 64 mg/kg (23, 58). Mice subjected to modafinil at 75 mg/kg do not undergo locomotor sensitization with repeated doses (55), whereas mice subjected to modafinil at 150 mg/kg do. Cross-sensitization (wherein repeated administration of one agent potentiates the locomotor response to acute administration of another), is taken as indirect evidence that two agents act on a similar neurobiological substrate. Mice subjected to repeated administration of modafinil exhibit potentiated locomotor responses to acutely administered methamphetamine (59). Likewise, mice subjected to repeated administration of methamphetamine exhibit potentiated locomotor responses to acutely administered modafinil (59). While not mechanistic, these cross-sensitization studies suggest that modafinil and amphetamines share a common, or at least overlapping, neurobiological substrate. Repeated administration of the D1/D2 receptor agonist apomorphine cross-sensitizes rats to modafinil (58); this fact provides further support for the notion that the relevant substrate is dopaminergic transmission.

The concept that modafinil acts via DAT inhibition might be regarded as controversial because of inconsistencies in the preclinical and clinical literature on the potential for abuse and addiction. The purpose of this article is not to review exhaustively the addictive potential of modafinil; this topic is covered elsewhere from a clinical perspective (60, 61). Rather, a brief survey of the pertinent literature serves to illustrate why differences between modafinil and other DAT-binding agents in putative measures of abuse potential does not nullify DAT binding as the mechanism of action for modafinil.

There is some evidence, from preclinical models purported to measure the potential for abuse and addiction, that modafinil has rewarding properties. For example, modafinil has discriminative stimulus effects in animals trained to engage in operant behavior when exposed to cocaine. This effect of modafinil has been documented in rodents (12, 54, 62), rhesus monkeys (63, 64), and humans (60). In mice, R-modafinil and S-modafinil were equipotent in discriminative stimulus assays (12), which, like microdialysis data mentioned above, leaves in question the significance of the enantiomeric differences in DAT-binding pharmacology. Modafinil also has a modest (relative to methylphenidate) discriminative stimulus effect in rats trained to engage in operant behavior when exposed to d-amphetamine (65). Drug-naïve mice exposed to modafinil at 75 (55) or 125 mg/kg (66) exhibited conditioned-place preference (CPP), a behavioral gage of reward that also has been found to be induced by cocaine [for instance, (55)]. However, this finding was not replicated in rats. Rats exhibited either no place preference [64 mg/kg; (67)] or a significant aversion to the environment in which they had previously been exposed to modafinil [32 or 64 mg/kg; (23)]. Furthermore, modafinil does not promote the reinstatement of cocaine self-administration (68) or methamphetamine-seeking (69–71) in rats.

Data from human subjects discriminate modafinil from cocaine in terms of abuse potential. Cocaine users do not report a high when exposed to modafinil (72, 73); rather, they report that modafinil blunts the subjective effect of cocaine when the two drugs are administered simultaneously (74, 75). One clinical trial reported that modafinil increased the maximum number of consecutive days of cocaine abstinence across a 12-week clinical trial. However, at the end of that 12-week trial there was no evidence of a decrease in total days of abstinence (76). And other clinical trials in humans, in which modafinil has failed to significantly improve abstinence rates during methamphetamine withdrawal (77, 78) or cocaine withdrawal (79), are revealing from both a conceptual standpoint and a practical one. If modafinil were a pharmacological mimetic of either of these agents, presumably it would substitute more effectively and promote sustained abstinence from the original agent. Therefore, although there are conflicting data in the literature, the majority of both preclinical and clinical data suggest that modafinil is pharmacologically distinct from both cocaine and amphetamines in the context of abuse and addiction.

Thus, ambiguities in the literature on drug abuse and sleep contribute to the concept that modafinil is somehow novel and distinct from amphetamines and cocaine. Notwithstanding the fact that modafinil, cocaine, and amphetamines all interact with DAT, the pharmacology of modafinil is distinct from that of cocaine and amphetamines. Whereas cocaine (80) and amphetamines (49) bind to the DA transporter with nanomolar affinity, modafinil acts as a DAT ligand at micromolar concentrations (10, 14). This low affinity may contribute to the slow kinetics of its effect on extracellular DA concentration (discussed below). Considering the monoamine transporters DAT, NET, and SERT, modafinil is highly selective as a DAT ligand (10, 12, 13, 18), albeit at micromolar concentrations. Cocaine (80) and amphetamines (49) disrupt the function of all three of the cell-membrane monoamine transporters. Additionally, both cocaine and amphetamines disrupt the vesicular monoamine transporter that packages monoamines into vesicles within the cell (81). Whether modafinil does so remains an open question.

The physiological relevance of the multi-transporter mechanism in responses to cocaine is illustrated by the failure of genetic inactivation of any one transporter to nullify the behavioral effects of cocaine. Neither DAT knockout (82) nor dual NET/SERT knockout (83) eliminated cocaine-induced CPP. A 90% reduction of DAT expression, induced by modifications of promoter sequences in the gene, was also not sufficient to disrupt cocaine-induced locomotion or CPP (84). These observation were taken as support for the concept that the rewarding effects of cocaine are mediated by multiple transporters. However, subsequent analyses with more sophisticated models led to a more complicated story. DAT knockout mice exhibited self-administration of cocaine only transiently and at reduced frequency relative to wild-type controls (85). A mouse genetic model was engineered in which DAT is expressed at normal levels but is modified in sequence such that it 89-fold less sensitive to inhibition by cocaine (86). These mice failed to exhibit cocaine-induced operant behavior (conditioned-place preference) and exhibited a decrease in locomotor activity in response to cocaine. These data indicate that (a) DAT mediates rewarding effects of cocaine and (b) while cocaine has pharmacological effects on other monoamine transporters, it does not promote reward or locomotor activity through those transporters unless DAT is genetically inactivated. The effects of cocaine on multiple transporters are in direct contrast to modafinil, the wake-promoting effect of which is abolished in DAT-deficient mice (17). The selectivity of modafinil as a DAT inhibitor is pertinent to the treatment of narcolepsy. Modafinil is distinguished from amphetamines by its lack of efficacy in treating cataplexy in narcolepsy (19). Amphetamines and other agents that block uptake by NET are effective anti-cataplectic agents (21).

Modafinil is further distinguished from amphetamines and cocaine by virtue of the physical nature of its interactions with DAT. As a neurotransmitter sodium symporter, DAT undergoes a sequence of conformational changes in the process of transporting its ligand into the cell (87). The sequence begins when extracellular sodium promotes the assumption of an open-to-out (also known as outward-facing) conformation, which primes the transporter for ligand binding. Ligand binding causes a shift to the closed-to-out (also known as occluded) conformation. The presence of additional ligand molecules in the extracellular milieu promotes a shift from the closed-to-out conformation to the open-to-in (also known as inward-facing) conformation, which releases the bound ligand into the cytoplasm and frees the transporter to repeat this sequence of changes.

In the context of this sequence of conformational changes, DAT inhibitors can behave very differently. Those that exhibit abuse potential, such as cocaine, facilitate the open-to-out conformation. By contrast, those that do not exhibit abuse potential, such as GBR12909, facilitate the closed-to-out or open-to-in conformation. The classification of compounds into these two categories (“cocaine-like” vs. “atypical” inhibitors) can be ascertained in site-directed mutagenesis studies: point mutations that cause the transporter to preferentially adopt an open-to-out conformation increase the IC_50_ value of cocaine at DAT, but not that of atypical inhibitors, by 200-fold. According to this type of analysis, modafinil is an atypical DAT-binding agent (11), and this distinction from cocaine is true for both R- and S-modafinil assayed independently (12). Therefore, the relatively low abuse potential attributed to modafinil may reflect the nature of its interaction with DAT, not the absence of an interaction with DAT. The relationship between DAT conformation and abuse potential is admittedly a relatively new concept. Why the physicochemical nature of binding influences abuse potential is uncertain, but this emerging line of work offers a potential explanation for the relatively low abuse potential associated with modafinil without requiring some putative unknown mechanism.

Amphetamines are rather complex modulators of monoaminergic function. Consideration of their effects on dopaminergic cells distinguishes them from modafinil. Amphetamines are a substrate for monoamine transporters (88) and are imported into the cell (14). In so doing, they promote the reverse transport of other DAT substrates [^3^H]1-methyl-4-phenylpyridinium (14) and DA itself (89) out of the cell via the DAT. The distinct mechanisms of modafinil and amphetamines in the cell are illustrated by their pharmacological interactions: amphetamine-induced [^3^H]1-methyl-4-phenylpyridinium efflux is abolished by co-administration of modafinil with amphetamines (14). Amphetamines are further distinguished from modafinil by the fact that they act as agonists for the trace amine-associated receptor 1 (TAAR1) (89). Modafinil exhibits no activity as a TAAR1 agonist at physiologically relevant concentrations [below 100 micromolar; (15)]. TAAR1 activation promotes wakefulness (90) and simultaneously increases protein kinase C (PKC) activity *in vitro* (91). One effect of amphetamine exposure *in vivo* is the phosphorylation of known PKC targets (92), although this effect has yet to be linked to TAAR1 activation, specifically. PKC activation via direct TAAR1 stimulation may therefore contribute to methamphetamine-induced hypersomnolence. It is possible that this unique property of amphetamines relative to modafinil underlies, at least in part, their distinct effects on sleep-wake cycles. In this context, it would be informative to measure the impact of wakefulness induced by selective TAAR1 receptor ligands on subsequent sleep and to determine whether TAAR1 knockout alters the course of sleep-wake cycles subsequent to methamphetamine-induced wakefulness.

Finally, there are pharmacokinetic differences between modafinil and other DAT-binding agents. The timing of peak plasma levels and the plasma half-life of orally administered racemic modafinil in humans are twice those of methylphenidate (93). Half-life is enantiomer-specific: the half-life of R-modafinil [15 h at an oral dose of 50–400 mg; (94)] is threefold greater than that of S-modafinil (3). Peak plasma levels are achieved much more rapidly with nasally administered cocaine (95), smoked cocaine (96), or orally administered methylphenidate (93). These agents yield peak plasma concentrations within an hour of administration. The pharmacokinetic difference between modafinil and cocaine is accompanied by a difference in the temporal profile of the effects of these compounds *in vivo* on extracellular DA in the nucleus accumbens in mice. Whereas cocaine caused extracellular DA concentrations to increase to peak levels by 30 min and decrease to less than half of peak values within an hour, modafinil-induced elevation of extracellular DA did not peak until approximately 1 h after administration and remained at peak values until the experiment was terminated at 6 h (12). Table 2 summarizes the similarities and differences between modafinil and classical stimulants discussed in the preceding section.

**Table 2:** **Effects of modafinil compared to classical stimulants**.

	Modafinil profile similar to classical stimulants	Modafinil profile different from classical stimulants	Comment
Preclinical models of abuse potential	✓	✓	Conflicting data in preclinical literature (14, 54, 55, 57, 62–64, 66, 69–71)
Human studies on abuse potential/addiction		✓	Modafinil does not appear to have reinforcing effects in humans, blunts the subjective effects of cocaine when co-administered, does not improve abstinence rates during cocaine or methamphetamine withdrawal (72–75, 77–79)
Effects on sleep characteristics		✓	Modafinil-induced wakefulness does not cause acute rebound hypersomnolence (6, 45)
Treatment of cataplexy (human and animal models)		✓	Modafinil is ineffective in treating cataplexy in preclinical models and in patients with narcolepsy, whereas amphetamines are effective anti-cataplectic agents (19–22)
Physical interaction with the DAT		✓	Modafinil facilitates a different conformation compared to cocaine; in contrast to amphetamine, modafinil does not reverse the transporter (11, 14)
Human pharmacokinetics after administration		✓	Modafinil reaches peak plasma levels in 2–4 h compared to 1 h for classical stimulants (93, 95)
Effects on DA concentration *in vivo*		✓	Modafinil takes longer to achieve peak extracellular concentrations of DA and elevations in extracellular DA levels are maintained longer (12)
Affinity for other monoamine transporters		✓	Modafinil has very low affinity for NET and no affinity for SERT; classical stimulants have nanomolar affinity for all three monoamine transporters (10, 12, 18, 13, 49, 80)
Affinity for TAAR1		✓	Modafinil shows no activity at TAAR1, whereas amphetamine acts as an agonist (15, 89)

DA, dopamine; DAT, dopamine transporter; NET, noradrenaline transporter; SERT, serotonin transporter; TAAR1, trace amine-associated receptor 1.

The relative reinforcing efficacy of DAT-binding agents is inversely proportional to their pharmacokinetic profiles: those compounds that are rapidly absorbed into and rapidly cleared from the system exhibit more addictive potential than those with slower kinetics (97–99). Therefore, putative differences in addictive potential and perceived rewarding effects between modafinil and DAT-binding agents such as cocaine, methamphetamine, and methylphenidate do not require the invocation of distinct sites of action.

## Conclusion

Parsimony dictates that distinct effects on clinically relevant measures be attributed to the known pharmacological differences between modafinil and other DAT-binding compounds, rather than to occult, unknown effects of modafinil at sites other than DAT. Modafinil elevates extracellular DA concentrations by binding to and disrupting the activity of the cell-membrane DAT. The resulting elevation of extracellular DA, through increased stimulation of dopaminergic and adrenergic receptors, results in wakefulness. Other signaling mechanisms that have been implicated in the response to modafinil are likely to be secondary to its catecholaminergic effects. While modafinil can be distinguished from other catecholaminergic agents in terms of clinical endpoints such as post-treatment hypersomnolence and its potential for addiction and abuse, these differences may be due to differences in selectivity for DAT, pharmacokinetics, or distinctions in the physicochemical nature of their interactions with the DAT. Additional work might clarify the exact basis for these subtle distinctions among wake-promoting agents. Still, the preponderance of information on the mechanism of action of modafinil points us in the direction of the DAT.

Future efforts to understand and therapeutically exploit wake-promoting mechanisms might better be directed toward more innovative strategies. The use of ligands for histamine H_3_ receptors (100), hypocretin receptors (101), or TAAR1 receptors (90) for instance, to ameliorate potential cognitive deficits secondary to sleep loss, is a relatively novel and potentially impactful strategy. The application of non-pharmacological approaches to treat performance deficits secondary to sleep insufficiency also has potential. In the context of narcolepsy, regenerative therapies for the hypocretin system, which undergoes degeneration in narcolepsy, are worthy of attention and effort (102, 103). In addition, transcranial manipulation of the electrical activity of the cerebral cortex (ultimately the seat of fatigue-related deficits) for therapeutic intervention is fast becoming a reality (104, 105).

## Conflict of Interest Statement

The author declares that the research was conducted in the absence of any commercial or financial relationships that could be construed as a potential conflict of interest.
